# Supplementary material for: Mint essential oil: A natural and effective agent for controlling house dust mites
Source: PLoS One. 2025 Feb 6;20(2):e0318639. doi: 10.1371/journal.pone.0318639 (PMC11801550; doi:10.1371/journal.pone.0318639)
Supplement: Attachment — Submitted filename: pone.0318639.docx [file pone.0318639.s003.docx]

Respond to comments

Comments 1. Please remove any funding information related text in the manuscript.

Respond: Done! We had remove the funding information.

Comments 2. If possible, please upload a file showing your changes either highlighted or using track changes. This should be uploaded as a Revised Manuscript w/tracked changes, file type.

Respond: No problem! We had submitted the change traced version.
