# Supplementary material for: Mint essential oil: A natural and effective agent for controlling house dust mites
Source: PLoS One. 2025 Feb 6;20(2):e0318639. doi: 10.1371/journal.pone.0318639 (PMC11801550; doi:10.1371/journal.pone.0318639)
Supplement: Attachment — Submitted filename: Response to Reviewers Comments - Komivi Akutse.docx [file pone.0318639.s001.docx]

**Point-by-point responses to Reviewers and Editor**

**Reviewer #1: Comments:**

Introduction:

L47-48 – needs citation.

Respond: The citation is included as suggested.

L50 – capitalization not required for yellow mealworm larvae

Respond: Corrected.

L51-52- needs citation.

Respond: Citation included as suggested.

L52-54 – needs citation.

Respond: Done as suggested.

L69-73 – should this section be in past, or future tense – may depend on journal format?

Respond: The section has been revised accordingly.

Methods:

L76 – When starting a sentence, genus names should always be written in full and not abbreviated. Change elsewhere, including in figure titles.

Respond: Many thanks for this observation. This has been corrected throughout the manuscript as suggested.

L87 – Table 1 – Abbreviations for the mint varieties are useful for later context, however the images of the plants provide nothing informative to this study. Please remove them.

Respond: Revised as suggested.

L87 – Table 1 title should read ‘Ten varieties of mint used in this study and their respective abbreviations’.

Respond: Corrected as suggested.

L91 – change ‘A 1 kg mint sample’ to ‘Each one kg mint sample’.

Respond: Done as suggested.

L94 – list the supplier information for the sodium sulfate.

Respond: Supplier information provided as suggested.

L99 – provide address of manufacturer.

Respond: Provided as suggested.

L115 – Provide version and years for the spectral libraries used. What minimum confidence % was used to confirm identifications from NIST if there were no standards or Kovats indices used?

Respond: NIST 2017. It was added in the revised version of the manuscript to capture that as suggested.

L116 – title ‘Bioassay’ is vague. Change to ‘Acaricidal bioassay’

Respond: Done as suggested.

L123 – describe how was the sex and maturity of these mites determined.

Respond: We did not distinguish the sex of mites, and the maturity was determined by the exuviate time.

L125 – provide supplier information for benzyl benzoate.

Respond: Provided as suggested.

L133 – remove ‘the’

Respond: Deleted as suggested.

L137 – ‘approximately three times the contact+fumigant LC100 values for each compound’ – a table listing these would be useful (or possibly add this information to Table 3 which has LC50 values?

Respond: The information is now added to Table 3 as suggested.

L140 – how large was the hole in the lid? This is important as it impacts how much opportunity there will be for losing volatiles.

Respond: 0.5 cm diameter, and this is now added in the revised version of the manuscript.

L141 – How were these containers maintained during the experiment? Were they in a closed container? A fume hood? What were the airflow parameters, temperature and humidity? – all factors which will influence the rate of volatile.

Respond: Kept in a closed container and the information is now added in the revised version of the manuscript as suggested.

L148 – LC15 concentration should also be presented in a table for comparison between varieties.

Respond: Addressed as suggested, and this is now included in Table 3.

L151 – “After placing the growth medium on filter paper for 5 minutes, 30 adult mites (without food) remaining on the paper were transferred to the compound-treated section using a brush to avoid damage” – I find this confusing to visualize. Suggest adding an image of the set-up showing the position of treated cloth and how the mites are added.

Respond: We revised the statement for more clarity and easy to visualize.

L153-155 – were the petri dishes covered or sealed in any way – not indicated.

Respond: Yes, they were covered; this is now captured in the revised version of the manuscript to avoid confusion.

L155- The term ‘escape’ suggested that a subject has exited the arena, but I believe its being used here to indicate repellency. I suggest replacing with ‘repellency’ throughout, unless the author truly means the mites are leaving the petri dish.

Respond: Many thanks for this remark. Changed to repellency throughout the text as suggested.

L159 – Add a citation for Abbott’s formula.

Respond: Done as suggested.

L167 – What statistical package was used? What was the alpha value used?

Respond: Graphpad Prism 9.0 software were used and now captured in the revised version of the manuscript.

Results and Discussion:

L174 – Table 2. Title needs to be more descriptive and specific to this study. For example, instead of “Table 2. Main compound of 10 kinds of essential oils”, I suggest “Table 2: Most abundant compound identified from each of ten varieties of mint following steam distillation and GC-MS analyses”

Respond: Done as suggested.

L174 – Was there a blank solvent run of chloroform used to subtract the solvent front? Trying to determine how the % area calculations compensated for this.

Respond: No, we did not use blank solvent.

L174 - Compounds need not be capitalized.

Tables 3 and 4 – alpha value for statistics should be reported.

Respond: Corrected as suggested.

L175 – change ‘time’ to ‘times’

Respond: Changed as suggested.

L176 – Write ‘var.’ in full for a header. Also change in other locations if used in titles for tables and figures.

Respond: Done as suggested.

L192 – Table 3. I’m very concerned about the concentrations and volumes being reported for the LC50s and fumigant toxicities. It would be very useful to see the % mortality reported alongside these values (its challenging to consider any of the volumes being reported here being of realistic application – even 65ul/cm2 of MC10 would seem a massive amount of solvent for a small area).

Respond: Revised as suggested.

L201 – 200ml/cm2 of MC5 oil? Is this correct? Seems physically impossible.

Respond: Sorry for this mistake. That is 200 μl/cm^2^ and it is now corrected in the revised version of the manuscript.

L219 – ‘Repellent effect of branched fatty acids’? This has nothing to with this paper?

Respond: Sorry for this mistake from our end. This should rather read ‘Repellent effect of main compounds of mint’. It is now corrected in the revised manuscript.

L222-223 – “The absence of dead mites following ethanol treatment indicates that the repellent effect is attributed to the mint oils and main compounds”. Its unclear what this statement is trying to infer, but given that this is reporting a repellent assay, one would be typically using sublethal dosages. I’m also wondering if this bioassay should be names something different – since it actually investigates a range from 100% mortality to 0% repellency.

Respond: The section is revised accordingly for more clarity and avoid confusion.

L224 – see above comment on ‘escaping’

Respond: Addressed accordingly.

L230 – Suggest changing the mean number of mites repelled (20.33, 19.33, 17.33) to a percentage instead.

Respond: Corrected as suggested.

L234 – spelling “subletial’ = ‘sublethal’

Respond: Corrected.

L235 – “The strong aroma of mint may disrupt the sensory receptors

of house dust mites, deterring their presence and reducing infestation risks” – this is a very vague statement, and needs further context and explanation.

Respond: The sentence is now revised for more clarity.

References: - needs a thorough check for formatting consistency, as well as correct formatting for latin names

Respond: The references have been revised as per the guidelines of the journal for uniformity.

Figures:

- Indicate the LD50 on the figures.

Respond: Done.

Other points:

- The presentation and interpretation of the repellent bioassay is insufficient. More comparison and detail is warranted.

Respond: Done!

- The discussion should be expanded to include points on:

o Potential mode of action of the essential oil constituents (active ingredients) which were investigated. There are many papers which investigate the effects of these compounds (i.e. linalool, menthol, limonene) on other species. Discussing the known lethal and sublethal effects on other arthropods, and acarines, would be important for context.

Respond: Done!

o More detail on proposed application of this knowledge, or key next steps should be included.

Respond: Done!

**Reviewer #2:**

Lines 46–49: Please provide references for these statements.

Respond: Reference included as suggested.

Lines 51–54: Please provide references for these statements.

Respond: Done as suggested.

In table 1, what is “M. × piperita”?

Respond: That is *Mentha piperita.* It is now corrected in the revised version of the manuscript.

Extraction of Essential Oils; Please briefly explain the methods and materials used for essential oil extraction.

Respond: More explanation has been added as suggested (lines xxxxx).

Why did the author use 5-8-day-old adults for bioassay? What is the average adult longevity for this species? What is the ratio of males and females used in the bioassay?

Respond: According to Kim JR, Perumalsamy H, Kwon MJ, Chae SU, Ahn YJ. *Toxicity of hiba oil constituents and spray formulations to American house dust mites and copra mites*. Pest Manag Sci. 2015 May;71(5):737-43, the average of adult longevity of house dust mite is about 20 days! That is why we selected one week old adult for the bioassay for easy manipulation and avoid physical damage. We did not distinguish the sex for mite during the bioassays.

What concentration of mint oil was used for the experiment? How did the author prepare the stock solutions? The author needs to provide this information through materials and methods.

Respond: Various concentrations of each test substance in 50 μL of ethanol were administered to 4.5 cm diameter black cotton-fabric circles, resulting in mint oil quantities of 300, 150, 75, 37.5, 18.75, 9.325, and 5 μL/cm² applied on the fabric. So, these were the various concentrations used, and they are captured in the materials and methods section of the revised manuscript. We used the pure mint oil directly which was consider as the stock solution too.

Why was benzyl benzoate used as a positive control? What concentration was used for the bioassay? Please provide information about benzyl benzoate in the chemicals section.

Respond: Benzyl benzoate was an effective recommended acaricide, and it was used in many previous studies. This is now included in the revised version of the manuscript.

Please utilize the statistical analysis in Table 3 to see the significant differences in LC50 values among the varieties.

Respond: Many thanks for the observation. However, LC50 is an estimated data rather than a measured data, and it is an estimated value of several sets of repeated test data, which is generally generated and not used for statistical difference analysis.

Were there any significant differences in mortality between the varieties?

Respond: Not yet observed as per our results. However, we can design further experiments as per your comment to investigate further on this aspect.
